# Cholinergic Interneurons Amplify Corticostriatal Synaptic Responses in the Q175 Model of Huntington’s Disease

**DOI:** 10.3389/fnsys.2016.00102

**Published:** 2016-12-16

**Authors:** Asami Tanimura, Sean Austin O. Lim, Jose de Jesus Aceves Buendia, Joshua A. Goldberg, D. James Surmeier

**Affiliations:** ^1^Department of Physiology, Feinberg School of Medicine, Northwestern UniversityChicago, IL, USA; ^2^Department of Medical Neurobiology, Institute of Medical Research Israel—Canada, Faculty of Medicine, The Hebrew University of JerusalemJerusalem, Israel

**Keywords:** channelrhodopsin-2, parafascicular nucleus, glutamatergic transmission, A-type K^**+**^ current, persistent Na^**+**^ current, ranolazine, paired-pulse ratio, minimal stimulation

## Abstract

Huntington’s disease (HD) is a neurodegenerative disorder characterized by deficits in movement control that are widely viewed as stemming from pathophysiological changes in the striatum. Giant, aspiny cholinergic interneurons (ChIs) are key elements in the striatal circuitry controlling movement, but whether their physiological properties are intact in the HD brain is unclear. To address this issue, the synaptic properties of ChIs were examined using optogenetic approaches in the Q175 mouse model of HD. In *ex vivo* brain slices, synaptic facilitation at thalamostriatal synapses onto ChIs was reduced in Q175 mice. The alteration in thalamostriatal transmission was paralleled by an increased response to optogenetic stimulation of cortical axons, enabling these inputs to more readily induce burst-pause patterns of activity in ChIs. This adaptation was dependent upon amplification of cortically evoked responses by a post-synaptic upregulation of voltage-dependent Na^+^ channels. This upregulation also led to an increased ability of somatic spikes to invade ChI dendrites. However, there was not an alteration in the basal pacemaking rate of ChIs, possibly due to increased availability of Kv4 channels. Thus, there is a functional “re-wiring” of the striatal networks in Q175 mice, which results in greater cortical control of phasic ChI activity, which is widely thought to shape the impact of salient stimuli on striatal action selection.

## Introduction

Huntington’s disease (HD) is an autosomal–dominant, progressive neurodegenerative disease associated with an expanded CAG repeat in the huntingtin (Htt) gene (Zuccato et al., 2010). Consistent with the prominent motor symptoms of the disease, there are clear pathological changes in the basal ganglia circuitry influencing goal directed movement and habit. In the striatum—the largest of the basal ganglia nuclei—principal spiny projection neurons (SPNs) atrophy (Deng et al., 2004). Recent work suggests that the atrophy of SPNs is due in part to impaired brain derived neurotrophic factor signaling through TrkB receptors (Canals et al., 2004; Plotkin et al., 2014).

Although mutant Htt (mHtt) profoundly affects SPNs (Deng et al., 2004; André et al., 2011) it also alters the properties of interneurons that modulate the striatal circuitry. Giant, aspiny cholinergic interneurons (ChIs) are key regulators of both SPN synaptic integration and intrinsic excitability (Kemp and Powell, 1971; Bolam et al., 1984; Wilson et al., 1990; Tepper and Bolam, 2004). Indices of striatal cholinergic signaling decline in the HD brain and in the brain of mouse models of the disease. Evoked acetylcholine (ACh) release is reduced (Farrar et al., 2011), as is the abundance of choline acetyltransferase, vesicular ACh transporters and muscarinic receptors (Cha et al., 1998; Smith et al., 2006). What is less clear is whether the physiological properties of ChIs are altered by mHtt in parallel.

ChIs are pacemaking neurons whose regular spiking is modulated by synaptic input (Bennett and Wilson, 1999). Both anatomical (Lapper and Bolam, 1992) and physiological (Ding et al., 2010; Threlfell et al., 2012; Doig et al., 2014) evidence suggests that although they receive cortical glutamatergic input, the intralaminar nuclei (ILN) of the thalamus provide the predominant source of extrinsic excitatory input to ChIs (Sidibe and Smith, 1999; Ding et al., 2010). This feature of ChIs might be critical to the pathophysiology of HD. In the disease, ILN neurons are lost (Heinsen et al., 1996; Kassubek et al., 2005). In the Q140 “knockin” mouse model of HD, there are fewer large axodendritic thalamostriatal terminals at presymptomatic ages (Deng et al., 2013), reportedly due to a reduction in dendritic branching in ChIs (Deng and Reiner, 2016). If the thalamic innervation of ChIs is reduced, their activation by behaviorally relevant stimuli, which depends upon the ILN, could be impaired (Matsumoto et al., 2001). This could have a wide range of deleterious effects on the striatal processing of movement related signals, including those arising from the cerebral cortex (Ding et al., 2010).

The studies described here examined the physiological properties of ChIs in presymptomatic (2–6 month old) Q175 heterozygous mice. It was found that thalamostriatal synaptic transmission to ChIs was altered in Q175 tissue, losing its facilitating response to repetitive stimulation. In addition, ChIs became significantly more responsive to strong optogenetic activation of corticostriatal inputs. The amplification of the corticostriatal synaptic currents was mediated by a post-synaptic, upregulation of voltage-dependent Na^+^ channels. This adaptation was apparently restricted to dendrites, as autonomous discharge of ChIs remained unchanged, whereas back-propagating action potentials (bAPs) reached more distal dendritic arbors in the ChIs from Q175 mice. Thus, our results suggest that there is a functional “re-wiring” of the networks controlling striatal ChI activity, which becomes more attuned to cortical input in HD.

## Materials and Methods

### Animals

This study was carried out in accordance with the recommendations of and approved by Northwestern University and the Hebrew University Animal Care and Use Committees. All of the experiments were conducted with 2–6 month old heterozygous Q175 male mice (Q175+/−) and their wildtype (WT) littermates on a C57Bl/6 background.

### Cortical Expression of ChR2

To investigate corticostriatal transmission to ChIs, homozygous transgenic mice [B6.Cg-Tg (Thy1-COP4/EYFP) 18Gfng/1] expressing channelrhodopsin-2 (ChR2) under the Thy1 promoter (Arenkiel et al., 2007) were crossed with Q175 mice (Menalled et al., 2003; Zuccato et al., 2010). In this particular line of Thy1-ChR2 mice, there is a robust expression of ChR2 in cortical pyramidal neurons, but little or no expression in thalamic nuclei projecting to the striatum, allowing optogenetic approaches to be used to study corticostriatal synaptic transmission.

### Thalamic Expression of ChR2

Bilateral stereotaxic injections into caudal ILN of thalamus were performed using standard approaches. AAV serotype 2/9 carrying fusion genes for hChR2 (H134R) and mCherry under CAG promoter (AAV-ChR2, University of Pennsylvania Vector Core, Addgene #20938MOD), were used to transfect parafascicular nuclei (PFN) neurons. Injection coordinates that target PFN (Ellender et al., 2013), which is thought to be the major source of thalamic inputs to ChIs (Lapper and Bolam, 1992; Ding et al., 2010; Threlfell et al., 2012), were identified using the Angle Two software (Leica, Buffalo Grove, IL, USA) and were from Bregma: lateral, 0.72 mm; posterior, 2.4 mm; and 3.5 mm depth from surface of brain. Briefly, stereotaxic surgeries were performed on mice anesthetized with isoflurane. A small hole was bored into the skull with a micro drill bit and a glass pipette was slowly inserted at the PFN coordinates. To minimize backflow, solution was slowly injected; a total volume of 0.1–0.3 μl ( >5 × 10^12^ GC) of the AAV constructs was injected over 1 min and the electrode was left in place for 10 min before slowly retracting it. Topical analgesics were applied to the skin around the suture (Lidocaine 2.5% and Prilocaine 2.5% cream) and subcutaneous injections of 1 mg/kg of 0.9% saline solution and analgesic Metacam 2% or Carpofen (5 mg/kg) were given. Two to 3 weeks after viral injections (which was an optimal time window for viral expression under the CAG promoter), mice were used for experiments.

### Slice Preparation

Animals were deeply anesthetized with ketamine–xylazine and perfused transcardially with ice-cold modified artificial cerebrospinal fluid (ACSF), bubbled with 95% O_2_-5% CO_2_, and containing (in mM): 2.5 KCl, 26 NaHCO_3_, 1.25 Na_2_HPO_4_, 0.5 CaCl_2_, 10 MgSO_4_, 0.4 ascorbic acid, 10 glucose, and 210 sucrose. The brain was removed and blocked in the sagittal plane and sectioned at a thickness of 275 μm in ice-cold modified ACSF. Slices were then submerged in ACSF, bubbled with 95% O_2_-5% CO_2_, and containing (in mM): 2.5 KCl, 126 NaCl, 26 NaHCO_3_, 1.25 Na_2_HPO_4_, 2 CaCl_2_, 2 MgSO_4_, and 10 glucose, and stored at room temperature for at least 1 h prior to recording.

### Slice Visualization, Electrophysiology and Optical Stimulation

The slices were transferred to the recording chamber mounted on an Olympus BX51 upright, fixed-stage microscope and perfused with oxygenated ACSF at 32°C. A 60×, 0.9 NA water-immersion objective was used to examine the slice using standard infrared differential interference contrast video microscopy. Patch pipette resistance was typically 3–4.5 MΩ when filled with recording solutions. In voltage-clamp experiments the intracellular solution contained (in mM): 127.5 CsCH_3_SO_3_, 7.5 CsCl, 10 HEPES, 10 TEA-Cl, 4 phosphocreatine disodium, 0.2 EGTA, 0.21 Na_2_GTP and 2 Mg_1.5_ATP (pH = 7.3 with CsOH, 280–290 mOsm/kg). In some experiments (e.g., paired-pulse ratio (PPR) measurements), the pipette contained 5 mM QX-314 bromide, as well. To record A-type hyperpolarization activated transient outward K^+^ currents the electrodes were filled with an intracellular solution containing (in mM): 135.5 KCH_3_SO_4_, 5 KCl, 2.5 NaCl, 10 HEPES, 0.2 EGTA, 5 phosphocreatine disodium, 0.21 Na_2_GTP and 2 Mg_1.5_ATP. For whole-cell current clamp recordings the pipette contained (in mM): 135.5 KCH_3_SO_4_, 5 KCl, 2.5 NaCl, 5 Na-phosphocreatine, 10 HEPES, 0.2 EGTA, 0.21 Na_2_GTP, and 2 Mg_1.5_ATP (pH = 7.3 with KOH, 280–290 mOsm/kg). For two-photon laser scanning microscopy (2PLSM) calcium imaging experiments the pipette contained (in mM): 135 KCH_3_SO_4_, 5 KCl, 5 Na-phosphocreatine, 5 Tris-phosphocreatine, 10 HEPES, 0.1 Fluo-4, 0.025 Alexa Fluor 568, 0.21 Na_2_GTP, and 2 Mg_1.5_ATP (pH = 7.3 with KOH, 280–290 mOsm/Kg).

Electrophysiological recordings were obtained with a Multiclamp 700B amplifier (Molecular Devices, Sunnyvale, CA, USA). Junction potential, which was 7–8 mV, was not corrected. Signals were digitized at 20–100 kHz and logged onto a personal computer with the Clampex 9.2 software (Molecular Devices). Blue light LED (470 nm, model M470L2, Thor Labs, Newton, NJ, USA or pE-100, CoolLED, Yorktown Heights, NY, USA) was used for full-field illumination via the objective (LED power was 1–19 mW). Single pulses were 1 ms long, pulse trains were 10 pulses long at 10 or 20 Hz.

### 2PLSM Imaging

The 2PLSM system was described previously (Guzman et al., 2010; Goldberg et al., 2012). Briefly, the two-photon excitation source was a Chameleon Ultra 2 tunable laser system (680–1080 nm; Coherent Laser Group, Santa Clara, CA, USA). Optical signals were acquired using an 810 nm excitation beam to excite Alexa and Fluo-4 dyes simultaneously. Laser power attenuation was achieved with two Pockels cells electro-optic modulators (models 350–50 and 350–80, Conoptics, Danbury, CT, USA). The fluorescence emission was collected with non-descanned photomultiplier tubes (PMTs; Prairie Technologies, Madison, WI, USA). A Dodt contrast detector system was used to provide a bright-field transmission image in registration with the fluorescent images. Scanning parameters: pixel size was 0.08 μm, dwell time: 10 μs. At the end of the experiment Z-series were acquired with pixel size: 0.36 μm and dwell time: 6 μs (Day et al., 2008).

### Drugs and Reagents

All experiments (except for measurements of tetrodotoxin (TTX)-sensitive currents) were conducted with a cocktail of synaptic blockers for ACh and GABA receptors including (in μM): 10 atropine, 10 mecamylamine, 2 CGP 55845, 10 SR 95531 and 50 D-APV. The fast inactivating sodium current was antagonized with TTX (1 μM), the persistent (“late”) Na^+^ current was antagonized with 30 μM ranolazine (Bennett et al., 2000; Abrams et al., 2006) and Cav1 calcium channels were blocked with 5 μM isradipine. All drugs and reagents were acquired from Tocris (Ellisvile, MO, USA) or Sigma (St. Louis, MO, USA), except for TTX that was acquired from Alomone lab (Jerusalem, Israel). Fluo-4 (Invitrogen, Carlsbad, CA, USA) and Alexa 568 (Invitrogen) were used to fill ChIs.

### Data Analysis and Statistics

Data were analyzed and curve fitting was done using custom-made code in Matlab (Mathworks, Natick, MA, USA). The nonparametric two-tailed Wilcoxon rank-sum test was used for independent samples, and the nonparametric two-tailed Wilcoxon signed-rank test was used for matched samples. Boxplots represent range (whiskers), median (thick bar) and lower and upper quartiles. In the current-voltage and current-LED intensity curves the medians of each measurement are presented with confidence intervals given by the 50% × (1 ± 1/√*n*) percentiles, where *n* is the sample size (Lasser-Katz et al., in press). The parametric ANCOVA test was used to test significant changes in these curves. Null hypotheses were rejected if the *P*-value was below 0.05.

## Results

### Functional Properties of Thalamic Synapses on ChIs were Modestly Altered in Q175 Mice

**Figure 1:**
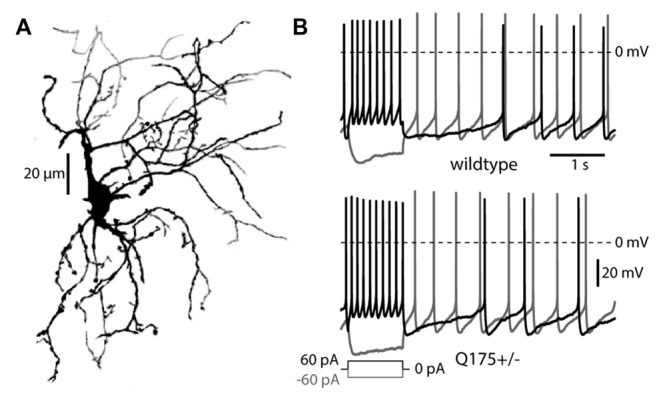
**Morphology and firing patterns of cholinergic interneurons (ChIs) in the Q175 mouse. (A)** Z-axis projection of an Alexa 568 filled ChI from a Q175+/− (heterozygous) mouse imaged with 2PLSM. **(B)** ChI responses to long depolarizing and hyperpolarizing pulses in Q175+/− and wildtype mice.

Recent work has suggested that the thalamic innervation of the striatum is reduced in HD models (Deng et al., 2013; Deng and Reiner, 2016). This innervation arises from the PFN of the thalamus (Lapper and Bolam, 1992; Consolo et al., 1996; Sidibe and Smith, 1999; Matsumoto et al., 2001; Bradfield et al., 2013). To study the impact of mHtt on this projection, the physiological properties of thalamostriatal synapses on ChIs in presymptomatic, 2–6 month old heterozygous Q175 (Q175+/−) and WT mice were examined. Visually identified ChIs invariably had prototypical morphological (Figure 1A) and electrophysiological features, including autonomous pacemaking, voltage sag in response to hyperpolarization, and slow afterhyperpolarizations following a long depolarization (Figure 1B; Kawaguchi, 1993; Bennett and Wilson, 1999). In these presymptomatic mice, there were no obvious morphological changes or alterations in basic electrophysiological properties of ChIs, in agreement with recent work in the R6/2 HD model (Holley et al., 2015).

**Figure 2:**
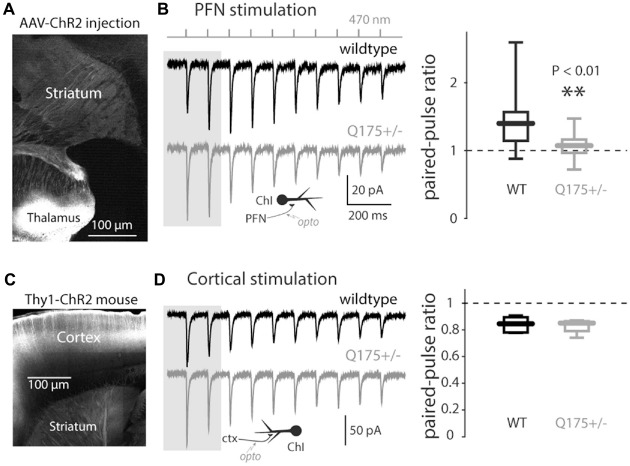
**Light-driven paired-pulse ratios (PPRs) of thalamo- and cortico-striatal synapses onto ChIs in the Q175 mouse. (A)** Sagittal section with the site of AAV-ChR2 transfection visible in the thalamus and axonal expression in the striatum. **(B)** Left: examples of trains of optogenetically induced thalamostriatal excitatory postsynaptic currents (EPSCs; 10 Hz) in wildtype (WT), (black trace) and Q175+/− (gray) mice. Inset: schematic of optogenetic stimulation of thalamostriatal axons; Right: boxplot of thalamostriatal PPR values in both genotypes (measured using the first two EPSCs in the train as illustrated by the gray background). **(C)** Sagittal cortical section in Thy1-ChR2 mice. **(D)** Left: examples of trains of optogenetically induced corticostriatal EPSCs in WT mice. Inset: schematic of optogenetic stimulation of corticostriatal axons; Right: boxplot of corticostriatal PPR values in both genotypes.

To determine if there were functional changes in the thalamic innervation of ChIs, optogenetic approaches were used. Stereotaxic injection of AAV carrying a ChR2 expression construct into the PFN (Figure 2A) led to robust excitatory postsynaptic currents (EPSCs) in ChIs in response to optical stimulation (Ellender et al., 2013; Parker et al., 2016). As shown previously (Ding et al., 2010), these synaptic connections were facilitating (increasing in amplitude with repetition; Figure 2B). The degree of synaptic facilitation was measured by computing the PPR, which was defined as the ratio of the amplitude of the second EPSC divided by the first one. PPRs were significantly reduced in 4–6 month old Q175 mice (WT median: 1.40, *n* = 15 neurons, *N* = 3 mice; Q175+/− median: 1.07, *n* = 14 neurons, *N* = 3 mice, *P* < 0.01, two tailed Wilcoxon rank-sum test, Figure 2B). The aggregate strength of the PF innervation of ChIs could not be reliably assessed because of variability in the extent of PF infection by the AAV carrying the ChR2 expression construct.

### The Response to Strong Cortical Excitation was Elevated in Q175 ChIs

Glutamatergic pyramidal neurons in the cerebral cortex also innervate striatal ChIs (Lapper and Bolam, 1992; Sidibe and Smith, 1999; Thomas et al., 2000; Ding et al., 2010; Doig et al., 2014; Kosillo et al., 2016). To determine if corticostriatal synapses on ChIs were affected by mHtt, Q175 mice were crossed with a transgenic mouse expressing ChR2 throughout the cerebral cortex (Arenkiel et al., 2007). Unlike the situation with AAV-dependent ChR2 expression, this transgenic model yielded a consistent pattern and level of ChR2 expression. Moreover, it allowed spatially distributed cortical inputs to ChIs to be reliably stimulated. In these mice, the yellow fluorescent protein (YFP) reporter of ChR2 expression was absent in the thalamus, but was robust in the cortex (Figure 2C). Optogenetic approaches were used to activate cortical axons in *ex vivo* brain slices while recording from ChIs. As suggested previously, the cortically PPR in ChIs was depressing (Ding et al., 2010). This relationship was unchanged in 3–6-month old Q175 mice (WT median: 0.85, *n* = 7 neurons, *N* = 2 mice; Q175+/− median: 0.86, *n* = 4 neurons, *N* = 1 mouse, *P* > 0.75, two tailed Wilcoxon rank-sum test, Figure 2D), suggesting that there was no alteration in glutamate release probability from cortical terminals in Q175 mice.

**Figure 3:**
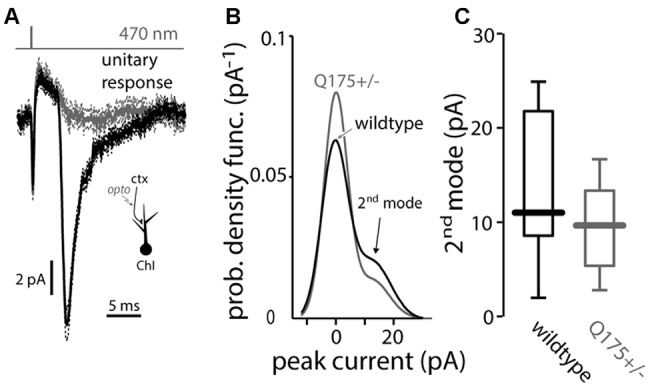
**Light-driven minimal stimulation of cortico-striatal synapses onto ChIs in the Q175 mouse. (A)** Measurement of unitary EPSCs using a minimal stimulation protocol where the presence (black traces) or absence (gray) of a response is stochastic (note the compound action potential due to synchronous activation of ChR2-laden corticostriatal axons that is visible in both cases). **(B)** Empirical probability density function of the unitary EPSC amplitude is fit by a binomial mixture-of-Gaussians model of the minimal synaptic responses and failures in wildtype (black trace) and Q175+/− (gray trace) mice. **(C)** Boxplot of the value of the 2nd mode of the mixture in both genotypes.

Minimal local optical stimulation was used to evoke unitary EPSCs in ChIs (Higley et al., 2009). The probability density function of the response amplitude was estimated and then fit with the sum of two Gaussian distributions (Redman, 1990). The fits to the 4-month old WT and Q175 mice were very similar, sharing a 2nd mode, indicating that the individual synaptic responses were also unchanged in the Q175 ChIs (WT median: 11.0 pA, *n* = 7 neurons, *N* = 2 mice; Q175+/− median: 9.6 pA, *n* = 8 neurons, *N* = 2 mice, *P* > 0.6, two tailed Wilcoxon rank-sum test, Figure 3).

**Figure 4:**
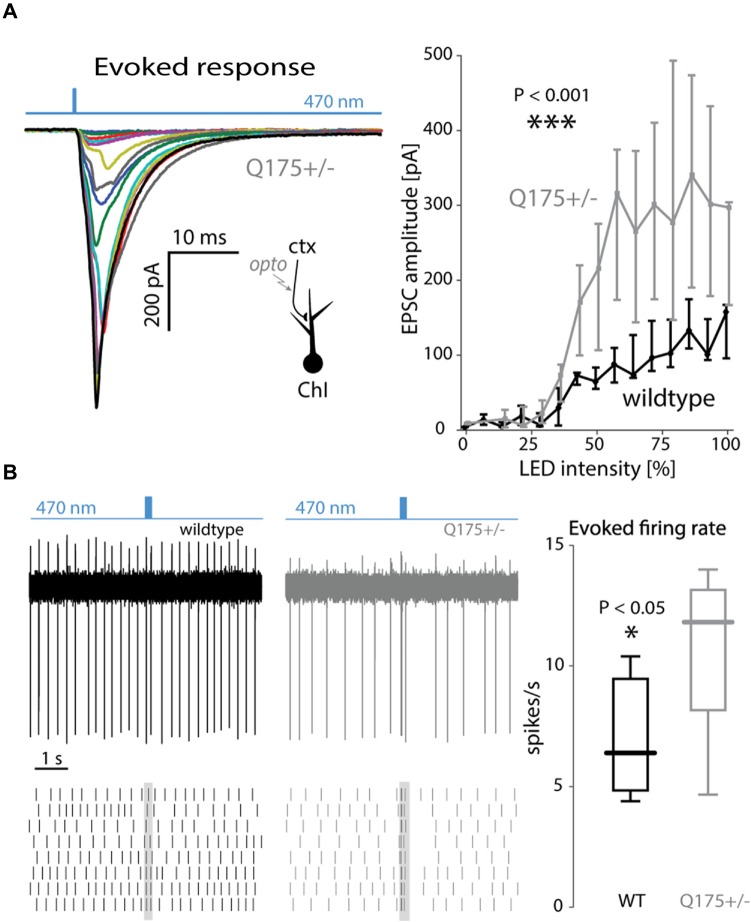
**Strong cortical stimulation augmented EPSCs and excitations of ChIs in the Q175 mouse. (A)** Left: EPSCs of increasing size in response to increasing LED intensity. Right: summary graph of EPSC amplitude as a function of LED intensity in wildtype (WT) (black trace) and Q175+/− (gray trace) mice. **(B)** Left: example trace and raster plot of spiking responses in ChIs to trains (20 Hz) of LED pulses in WT (black) and Q175+/− (gray) mice. Right: boxplot of amplitude of evoked spiking responses in ChIs (measured during the duration of the LED pulse train) in both genotypes.

Next, the input-output relationship of the cortical response in ChIs was estimated using light pulses of increasing intensity (Figure 4A). As the light intensities grew, the EPSC amplitude in ChIs from 2 to 3-month old Q175 mice rose more than in WT littermates (WT: *n* = 7 neurons, *N* = 3 mice; Q175+/−: *n* = 6 neurons, *N* = 3 mice, *P* < 0.001, ANCOVA, Figure 4A). The elevation in Q175 ChI responses to cortical stimulation also was evident when a short burst of optical stimuli (20 Hz, 10 pulses) was used and the evoked discharge rate measured in cell-attached mode. The enhanced synaptic response led to an increase in spiking. In ChIs from 4-month old WT mice, bursts of cortical activity only modestly increased the probability of ChI spiking, whereas in ChIs from Q175+/− mice, the same stimuli evoked robust spiking (WT median: 6.4 spikes/s, *n* = 7 neurons, *N* = 4 mice; Q175+/− median: 11.8 spikes/s, *n* = 7 neurons, *N* = 3 mice, *P* < 0.05, two tailed Wilcoxon rank-sum test) that was often followed by a pause in activity, as previously described to occur following thalamic stimulation (Ding et al., 2010; Figure 4B).

### Nav1 Channels were Necessary for Amplification of Cortical Input

**Figure 5:**
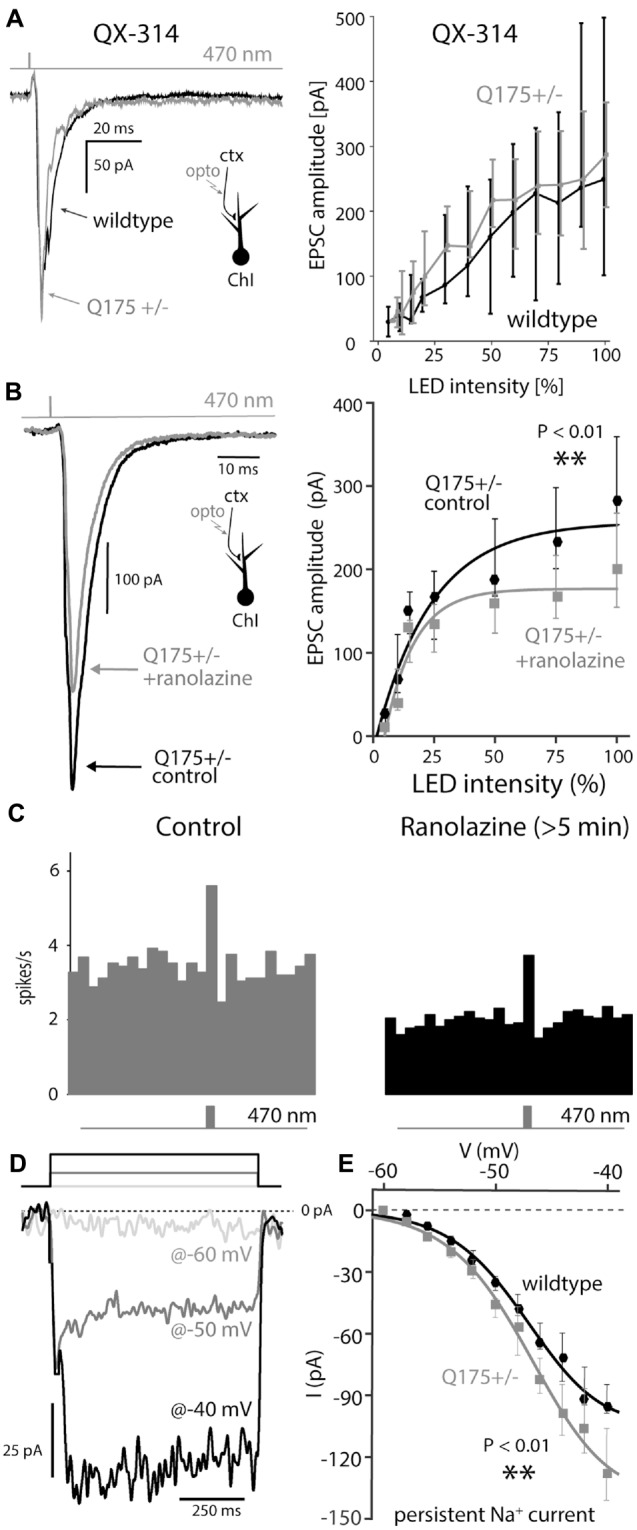
**Boosting of cortical input to ChIs depends of post-synaptic Nav1 channels that are upregulated in Q175 mice. (A)** Examples of EPSCs and dependence of their amplitude on LED intensity measured with 5 mM QX-314, a membrane impermeable Nav channel antagonists, in the recording pipette in wildtype (WT, black) and Q175+/− (gray) mice. **(B)** Example of a light-driven EPSC and dependence of their amplitude on LED intensity before (black) and after (gray) ranolazine (30 μM). **(C)** Population peri-stimulus time histogram before (gray) and at least 5 min after (black) application of ranolazine. **(D)** Measurement of persistent TTX-sensitive inward currents in cesium loaded ChIs, in response to 800-ms-long voltage pulses (to −60 mV, −50 mV and −40 mV, color coded). **(E)** Persistent Nav1 current as a function of voltage in both genotypes.

The enhanced cortical response in Q175 ChIs was not dependent upon NMDA receptors, as all EPSC measurements and spiking responses were conducted while bathing the slices in the NMDA receptor antagonist D-APV (50 μM). We therefore hypothesized that Nav1 channels could give rise to the amplification, as in neocortical pyramidal neurons (Schwindt and Crill, 1995). Indeed, when QX-314 (a membrane impermeable antagonist of Nav1 channels) was included in the pipette, the EPSC amplitudes in ChIs from 2 to 3-month old Q175 mice normalized and were no longer distinguishable from those evoked in WT mice (WT: *n* = 6 neurons, *N* = 2 mice; Q175+/−: *n* = 6 neurons, *N* = 2 mice, *P* > 0.65, ANCOVA, Figure 5A).

Another inward conductance in the same voltage range in ChIs that may be susceptible to QX-314 block is attributable to Cav1.3 Ca^2+^ channels (Yan and Surmeier, 1996; Goldberg and Wilson, 2005; Goldberg et al., 2009); however, the Cav1 channel antagonist isradipine (5 μM) had no effect on EPSC amplitude (data not shown). To further assess whether Nav1 channels were responsible for amplification of cortical inputs in ChIs, optically evoked EPSCs were measured in the presence of ranolazine, which preferentially antagonizes Nav1 channels in the persistent gating mode (Antzelevitch et al., 2004; Abrams et al., 2006). Bath application of ranolazine (30 μM) significantly reduced the amplitude of cortical EPSCs evoked by strong stimulation in 4-month-old Q175 mice (WT median: 284 pA; Q175+/−, ranolazine median: 200 pA, *n* = 8 neurons, *N* = 3 mice, *P* < 0.01, two tailed Wilcoxon signed-rank test, Figure 5B). Moreover, ranolazine reduced the ability of cortical stimulation to evoke spikes. In cell-attached mode, the spiking elicited by optical stimulation of corticostriatal axons was reduced relative to that seen prior to ranolazine application (Figure 5C), demonstrating that reducing the persistent-Nav1 dependent boosting of synaptic input translated into a reduced spiking response to cortical activation.

To determine if Nav1 channel currents were greater in Q175 ChIs, neurons were patch clamped using an internal solution containing cesium to block K^+^ channels and improve voltage control of dendrites. Because transient Nav1 channel currents in the dendrites could not be clamped (even with a cesium internal solution), long (800 ms) voltage steps from –60 mV were delivered to measure persistent currents; TTX was then applied and the TTX-sensitive part of the current computed by subtraction (Figure 5D; Bennett et al., 2000). Persistent Na^+^ currents measured in this way were significantly larger in ChIs from 2 to 3-month old Q175 mice than in WT littermates ; at –40 mV, the currents in Q175 ChIs were about 25% larger than those in wildtype ChIs (wildtype: *n* = 14 neurons, *N* = 2 mice; Q175+/−: *n* = 15 neurons, *N* = 3 mice, *P* < 0.01, ANCOVA, Figure 5E).

**Figure 6:**
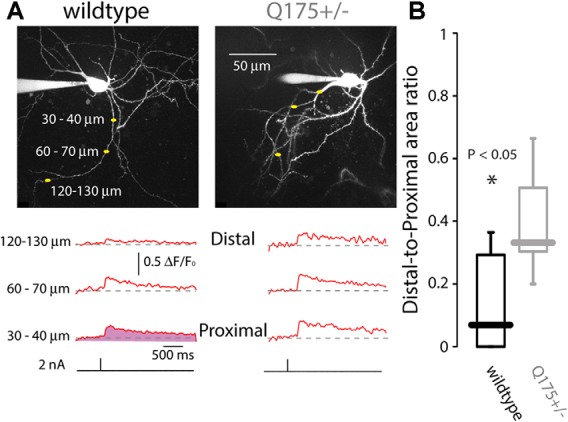
**Back-propagating action potentials (bAPs) infiltrate further into distal dendrites of ChIs in Q175 mice, as measured by 2PLSM Ca^2+^ imaging. (A)** Dendritic Ca^2+^ transients elicited by brief 2 nA somatic current pulses measured at three distances from the soma in wildtype (WT) and Q175+/− mice beneath 2PLSM Z-stacks of the recorded cells. **(B)** Boxplot of the area beneath the ΔF/F traces (e.g., depicted in the shaded area in bottom left trace) at the distal location divided by the area beneath the ΔF/F traces at the proximal location.

To provide an alternative test of whether dendritic Na^+^ channels were up-regulated in Q175 ChIs, the ability of somatically generated bAPs to invade the dendrites was examined using 2PLSM and the Ca^2+^ dye Fluo-4 in *ex vivo* brain slices; in these experiments, Ca^2+^ entry through voltage-dependent Ca^2+^ channels was used as a surrogate measure of dendritic depolarization driven by Na^+^ channels. In ChIs from 2 to 3-month old WT mice, bAP-evoked Ca^2+^ transients were observed only in the proximal parts of the dendritic arbor. However, in ChIs from littermate Q175 mice, the bAP-evoked Ca^2+^ transients invaded distal dendrites (WT median ratio of areas under ΔF/F_0_ curves: 0.068, *n* = 13 neurons, *N* = 5 mice; Q175+/− median ratio of areas: 0.330, *n* = 5 neurons, *N* = 4 mice, *P* < 0.05, two tailed Wilcoxon rank-sum test, Figure 6), consistent with the proposition that there was an up-regulation in dendritic Nav1 Na^+^ channels.

### Pacemaking Rate was Unchanged in Q175 ChIs

**Figure 7:**
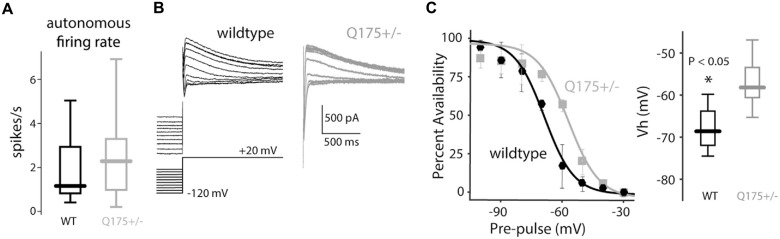
**Autonomous firing rate of ChIs is unchanged in the Q175 mouse. (A)** Boxplots of autonomous firing rates recorded in the cell-attached mode in wildtype (WT, black) and Q175+/− (gray) mice. **(B)** Representative traces of A-type K^+^ current recorded at +20 mV after a series of hyperpolarizing steps in WT (black trace) and Q175+/− (gray trace) mice. **(C)** Left: inactivation curves of A-type current in both genotypes. Right: boxplots of the half-inactivation voltage (Vh) in both genotypes.

Nav1 Na^+^ currents are necessary for pacemaking in ChIs (Bennett et al., 2000; Maurice et al., 2004), and as shown above (Figure 5C), antagonizing the persistent-Nav1 current indeed slows and eventually stops pacemaking. Hence, it is possible that the up-regulation in Nav1 channel currents (Figure 5E) could increase pacemaking rate. However, measurement of the autonomous *in vitro* firing rate of ChIs from 2 to 4-month old mice in the cell-attached mode (which preserves the intracellular milieu) revealed no difference between wild type and Q175 ChIs (WT median: 1.16 spikes/s, *n* = 17 neurons, *N* = 6 mice; Q175+/− median: 2.28 spikes/s, *n* = 32 neurons, *N* = 6 mice, *P* > 0.28, two tailed Wilcoxon rank-sum test, Figure 7A).

As pacemaking is readily seen in acutely dissociated ChIs that lack distal dendrites (Maurice et al., 2004), the ion channels responsible for its generation are localized to the proximal, somatodendritic compartment, including the axon initial segment. In this subcellular region, Kv4 K^+^ channels are known to make a major contribution to determining pacemaking rate (Song et al., 1998). Up-regulation in these channels could counteract the functional increase in Nav1 Na^+^ channel currents, leading to a normalization of pacemaking rate. To test this possibility, the properties of Kv4 channel currents were measured in ChIs (Figure 7B). While there was no difference in peak Kv4 channel currents or activation voltage-dependence, there was a significant rightward shift in inactivation voltage-dependence of Kv4 channels in Q175 ChIs (WT median half inactivation voltage: –68.6 mV, *n* = 4 neurons, *N* = 1 mouse; Q175+/− median half inactivation voltage: –58.2 mV, *n* = 5 neurons, *N* = 2 mice, *P* < 0.05, two tailed Wilcoxon rank-sum test, Figure 7C). This shift should increase outward Kv4 channel K^+^ window currents during pacemaking, counter-balancing the augmentation of inward Nav1 Na^+^ currents.

## Discussion

Three conclusions can be drawn from our studies about the striatal adaptations in presymptomatic Q175+/− mice. First, the autonomous spiking of ChIs was not significantly changed. Second, the release probability of thalamostriatal glutamatergic synapses to ChIs was altered. Third, the response of ChIs to strong stimulation of the cerebral cortex axons was enhanced; this enhancement was attributable to amplification of synaptic responses by post-synaptic voltage-dependent Nav1 channels. Taken together, these results suggest that the striatal circuitry is “re-wired” by mHtt, shifting control of ChI activity away from the thalamus and toward the cerebral cortex. This shift could have profound effects on the cholinergic modulation of the striatal circuit and movement control.

### Excitatory Synaptic Control of ChIs was Altered in HD Models

This reduction in the PPR of the thalamostriatal synapses onto ChIs is consistent with an increased release probability. This could be a compensation for a reduction in the total number of thalamostriatal synapses in the Q175 mouse (Deng et al., 2013; Deng and Reiner, 2016). There is evidence from human HD patients that ILN neurons are lost (Heinsen et al., 1996; Kassubek et al., 2005). However, because of variation in the infection of PFN neurons responsible for this projection, it was not possible to reliably determine whether there was in fact a reduction in the functional connectivity of ChIs with PFN in the Q175 model. Alternative strategies, like transgenic mice in which PFN neurons selectively express ChR2 or Cre recombinase, should allow this question to be definitively answered.

The clearest change observed was the amplification of Q175 ChIs responses to strong cortical stimulation. The enhanced responses originated in the postsynaptic membrane, as there was no discernible change in glutamate release probability or in the amplitude of unitary synaptic currents at cortical synapses. Amplification was seen only with strong synaptic depolarization that was capable of engaging voltage-dependent Nav1 Na^+^ channels and was eliminated by dialysis with QX-314, a lidocaine derivative that antagonizes Na^+^ channels (Strichartz, 1973) and by ranolazine, a selective antagonist of the “late” Na^+^ current (Abrams et al., 2006). Although Nav1 Na^+^ channel availability could not be directly estimated because of limitations in the ability of a somatic electrode to clamp rapidly gating dendritic channels, persistent Na^+^ current was larger in HD ChIs. Persistent Na^+^ current is due to a re-opening gating mode of Nav1 channels (Cantrell and Catterall, 2001; Carr et al., 2003); the molecular architecture of the Nav1 channel determines the probability of this gating mode, as different subunit combinations result in persistent Na^+^ current constituting from 1% to 5% of the peak transient current (Aman et al., 2009). The subunits contributing to Na^+^ currents in ChIs are heterogeneous, possibly reflecting differences in their localization within different subcellular compartments (Maurice et al., 2004). The lack of change in autonomous firing rates in the Q175 suggests that the upregulation of Na^+^ channels is not axosomatic. However, our finding that bAPs propagate farther into the dendritic arbor of the ChIs, supports the notion that the upregulation is largely dendritic, leading to amplification of cortical synapses located there.

### Functional Implications for the Pathophysiology of HD

There are several lines of evidence suggesting that hypocholinergic signaling in the striatum is a potentially important feature of early stage HD. A number of markers of cholinergic signaling—AChT, vACh, and MRs—are down-regulated in HD or models of HD (Enna et al., 1976; Wastek and Yamamura, 1978; Cha et al., 1998; Smith et al., 2006).

Our studies add a new dimension to this body of work, showing that the integration of excitatory synaptic inputs by ChIs is significantly altered in the Q175 model. Although it remains unclear whether the total synaptic input to ChIs from PFNs is altered, what was clearly different in the Q175 ChIs was their sensitivity to high frequency PFN spiking or bursting. Burst spiking in PFN occurs in response to behaviorally salient stimuli (Minamimoto and Kimura, 2002; Minamimoto et al., 2009), leading to enhanced activity in striatal indirect pathway SPNs (iSPNs) and suppression of movement. This “stop and look” response should be impaired in Q175 mice and in HD patients, potentially contributing to early stage hyperkinetic symptoms. Recent work demonstrating that GABAergic synaptic inputs to ChIs are up-regulated in the R6/2 model (Holley et al., 2015) provides a complementary mechanism by which the temporal pattern of ChI activity might be disrupted in HD.

An impairment in PFN activation of ChIs in response to salient or rewarding stimuli also could lead to deficits in synaptic plasticity. ChI controlled M1 muscarinic receptor stimulation is critical to long-term potentiation of glutamatergic synapses controlling appropriately timed and scaled iSPN activity necessary for movement suppression (Picconi et al., 2006; Wang et al., 2014). In direct pathway SPNs (dSPNs), phasic ChI activation of M4 muscarinic receptors counter-balances D1 dopamine receptor signaling (Onali and Olianas, 2002; Hernández-Flores et al., 2015), promoting long-term depression of cortical excitatory glutamatergic synapses (Shen et al., 2015). Thus, a deficit in thalamic PF control of ChIs could lead to attenuation of excitatory synapses on iSPNs and enhancement of excitatory synapses on dSPNs, again, contributing to the hyperkinetic symptoms characteristic of early stage HD.

What is less apparent is how amplification of cortical inputs to ChIs would alter network function. Recent mapping of cortical synaptic connections made on ChIs suggests that these arise primarily from cingulate cortex (unpublished observations). Like the ILN, cingulate cortex neurons are robustly activated by salient environmental events (Dean et al., 2004; Manza et al., 2016). Thus, the up-regulation of this input in HD models may be a compensatory response to the loss of ILN inputs (Heinsen et al., 1996; Kassubek et al., 2005; Deng et al., 2013; Deng and Reiner, 2016).

Our results and those of others suggest that inappropriately timed or scaled ChI activity could contribute to the hyperkinetic features of early stage HD. This insight opens several potential therapeutic avenues. Systemically administered M4 muscarinic receptor positive allosteric modulators (PAMs), which might partially compensate for diminished thalamic drive of ChIs in HD, have been shown to blunt unwanted movement evoked by levodopa treatment (Shen et al., 2015). Similarly, M1 muscarinic receptor PAMs might be effective in boosting event-driven ChI enhancement of iSPN activity, enabling suppression of unwanted activity. Alternatively, nicotinic receptor agonists might be effective in modulating striatal cholinergic signaling through GABAergic interneurons and dopaminergic terminals and in so doing suppress hyperkinetic symptoms in early stage HD (Quik et al., 2014).

### Summary

Our studies demonstrate that in presymptomatic Q175 mice, the functional connectivity between thalamic PFN neurons and ChIs is attenuated, resulting in amplification of cortical responsiveness. This amplification was due to amplification of dendritic voltage-dependent Nav1 channels. Thus, there is a functional “re-wiring” of the striatal networks in HD models, which results in greater cortical control of phasic ChI activity, which is widely thought to shape the impact of salient stimuli on striatal action selection.

## Author Contributions

AT conducted experiments and prepared a figure. SAOL and JJAB conducted experiments. JAG designed, directed and conducted experiments, prepared figures and wrote part of the manuscript. DJS designed and directed the experiments and wrote the manuscript.

## Funding

The CHDI Foundation (DJS), the JPB Foundation (DJS), a Marie Curie FP7 Integration Grant within the 7th European Union Framework Programme (no. PCIG13-GA-2013-630662; JAG), an Israel Science Foundation grant (no. 154/14, JAG), a European Research Council Consolidator grant (no. 646880-SynChI; JAG), the National Institute of Psychobiology in Israel—founded by the Charles E. Smith family (JAG), a Naiberg Family postdoctoral fellowship (JJAB), and a fellowship from Japan Society for the Promotion of Science Postdoctoral Fellow for Research Abroad (AT) funded this work.

## Conflict of Interest Statement

The authors declare that the research was conducted in the absence of any commercial or financial relationships that could be construed as a potential conflict of interest.

## References

[B1] Abrams J. Jones C. A. Kirkpatrick P. (2006). Ranolazine. Nat. Rev. Drug Discov. 5, 453–454. 10.1038/nrd2069 16821287

[B2] Aman T. K. Grieco-Calub T. M. Chen C. Rusconi R. Slat E. A. Isom L. L.. (2009). Regulation of persistent Na current by interactions between beta subunits of voltage-gated Na channels. J. Neurosci. 29, 2027–2042. 10.1523/JNEUROSCI.4531-08.2009 19228957 PMC2667244

[B3] André V. M. Cepeda C. Fisher Y. E. Huynh M. Bardakjian N. Singh S.. (2011). Differential electrophysiological changes in striatal output neurons in Huntington’s disease. J. Neurosci. 31, 1170–1182. 10.1523/JNEUROSCI.3539-10.2011 21273402 PMC3071260

[B4] Antzelevitch C. Belardinelli L. Wu L. Fraser H. Zygmunt A. C. Burashnikov A.. (2004). Electrophysiologic properties and antiarrhythmic actions of a novel antianginal agent. J. Cardiovasc. Pharmacol. Ther. 9, S65–S83. 10.1177/107424840400900106 15378132

[B5] Arenkiel B. R. Peca J. Davison I. G. Feliciano C. Deisseroth K. Augustine G. J.. (2007). *In vivo* light-induced activation of neural circuitry in transgenic mice expressing channelrhodopsin-2. Neuron 54, 205–218. 10.1016/j.neuron.2007.03.005 17442243 PMC3634585

[B7] Bennett B. D. Callaway J. C. Wilson C. J. (2000). Intrinsic membrane properties underlying spontaneous tonic firing in neostriatal cholinergic interneurons. J. Neurosci. 20, 8493–8503. 11069957 10.1523/JNEUROSCI.20-22-08493.2000 PMC6773196

[B6] Bennett B. D. Wilson C. J. (1999). Spontaneous activity of neostriatal cholinergic interneurons *in vitro*. J. Neurosci. 19, 5586–5596. 10377365 10.1523/JNEUROSCI.19-13-05586.1999 PMC6782311

[B8] Bolam J. P. Wainer B. H. Smith A. D. (1984). Characterization of cholinergic neurons in the rat neostriatum. A combination of choline acetyltransferase immunocytochemistry, Golgi-impregnation and electron microscopy. Neuroscience 12, 711–718. 10.1016/0306-4522(84)90165-9 6382048

[B9] Bradfield L. A. Bertran-Gonzalez J. Chieng B. Balleine B. W. (2013). The thalamostriatal pathway and cholinergic control of goal-directed action: interlacing new with existing learning in the striatum. Neuron 79, 153–166. 10.1016/j.neuron.2013.04.039 23770257 PMC3863609

[B10] Canals J. M. Pineda J. R. Torres-Peraza J. F. Bosch M. Martín-Ibañez R. Muñoz M. T.. (2004). Brain-derived neurotrophic factor regulates the onset and severity of motor dysfunction associated with enkephalinergic neuronal degeneration in Huntington’s disease. J. Neurosci. 24, 7727–7739. 10.1523/JNEUROSCI.1197-04.2004 15342740 PMC6729627

[B11] Cantrell A. R. Catterall W. A. (2001). Neuromodulation of Na^+^ channels: an unexpected form of cellular plasticity. Nat. Rev. Neurosci. 2, 397–407. 10.1038/35077553 11389473

[B12] Carr D. B. Day M. Cantrell A. R. Held J. Scheuer T. Catterall W. A.. (2003). Transmitter modulation of slow, activity-dependent alterations in sodium channel availability endows neurons with a novel form of cellular plasticity. Neuron 39, 793–806. 10.1016/s0896-6273(03)00531-2 12948446

[B13] Cha J. H. Kosinski C. M. Kerner J. A. Alsdorf S. A. Mangiarini L. Davies S. W.. (1998). Altered brain neurotransmitter receptors in transgenic mice expressing a portion of an abnormal human huntington disease gene. Proc. Natl. Acad. Sci. U S A 95, 6480–6485. 10.1073/pnas.95.11.6480 9600992 PMC27817

[B14] Consolo S. Baldi G. Giorgi S. Nannini L. (1996). The cerebral cortex and parafascicular thalamic nucleus facilitate *in vivo* acetylcholine release in the rat striatum through distinct glutamate receptor subtypes. Eur. J. Neurosci. 8, 2702–2710. 10.1111/j.1460-9568.1996.tb01565.x 8996820

[B15] Day M. Wokosin D. Plotkin J. L. Tian X. Surmeier D. J. (2008). Differential excitability and modulation of striatal medium spiny neuron dendrites. J. Neurosci. 28, 11603–11614. 10.1523/JNEUROSCI.1840-08.2008 18987196 PMC3235729

[B16] Dean H. L. Crowley J. C. Platt M. L. (2004). Visual and saccade-related activity in macaque posterior cingulate cortex. J. Neurophysiol. 92, 3056–3068. 10.1152/jn.00691.2003 15201314

[B17] Deng Y. P. Albin R. L. Penney J. B. Young A. B. Anderson K. D. Reiner A. (2004). Differential loss of striatal projection systems in Huntington’s disease: a quantitative immunohistochemical study. J. Chem. Neuroanat. 27, 143–164. 10.1016/s0891-0618(04)00028-6 15183201

[B18] Deng Y. P. Reiner A. (2016). Cholinergic interneurons in the Q140 knockin mouse model of Huntington’s disease: reductions in dendritic branching and thalamostriatal input. J. Comp. Neurol. 524, 3518–3529. 10.1002/cne.24013 27219491 PMC5050058

[B19] Deng Y. P. Wong T. Bricker-Anthony C. Deng B. Reiner A. (2013). Loss of corticostriatal and thalamostriatal synaptic terminals precedes striatal projection neuron pathology in heterozygous Q140 Huntington’s disease mice. Neurobiol. Dis. 60, 89–107. 10.1016/j.nbd.2013.08.009 23969239 PMC3808190

[B20] Ding J. B. Guzman J. N. Peterson J. D. Goldberg J. A. Surmeier D. J. (2010). Thalamic gating of corticostriatal signaling by cholinergic interneurons. Neuron 67, 294–307. 10.1016/j.neuron.2010.06.017 20670836 PMC4085694

[B21] Doig N. M. Magill P. J. Apicella P. Bolam J. P. Sharott A. (2014). Cortical and thalamic excitation mediate the multiphasic responses of striatal cholinergic interneurons to motivationally salient stimuli. J. Neurosci. 34, 3101–3117. 10.1523/JNEUROSCI.4627-13.2014 24553950 PMC3931511

[B22] Ellender T. J. Harwood J. Kosillo P. Capogna M. Bolam J. P. (2013). Heterogeneous properties of central lateral and parafascicular thalamic synapses in the striatum. J. Physiol. 591, 257–272. 10.1113/jphysiol.2012.245233 23109111 PMC3557661

[B23] Enna S. J. Bennet J. P. Jr. Bylund D. B. Snyder S. H. Bird E. D. Iversen L. L. (1976). Alterations of brain neurotransmitter receptor binding in Huntington’s chorea. Brain Res. 116, 531–537. 10.1016/0006-8993(76)90502-3 10053

[B24] Farrar A. M. Callahan J. W. Abercrombie E. D. (2011). Reduced striatal acetylcholine efflux in the R6/2 mouse model of Huntington’s disease: an examination of the role of altered inhibitory and excitatory mechanisms. Exp. Neurol. 232, 119–125. 10.1016/j.expneurol.2011.08.010 21864528

[B26] Goldberg J. A. Guzman J. N. Estep C. M. Ilijic E. Kondapalli J. Sanchez-Padilla J.. (2012). Calcium entry induces mitochondrial oxidant stress in vagal neurons at risk in Parkinson’s disease. Nat. Neurosci. 15, 1414–1421. 10.1038/nn.3209 22941107 PMC3461271

[B27] Goldberg J. A. Teagarden M. A. Foehring R. C. Wilson C. J. (2009). Nonequilibrium calcium dynamics regulate the autonomous firing pattern of rat striatal cholinergic interneurons. J. Neurosci. 29, 8396–8407. 10.1523/JNEUROSCI.5582-08.2009 19571130 PMC2739003

[B25] Goldberg J. A. Wilson C. J. (2005). Control of spontaneous firing patterns by the selective coupling of calcium currents to calcium-activated potassium currents in striatal cholinergic interneurons. J. Neurosci. 25, 10230–10238. 10.1523/JNEUROSCI.2734-05.2005 16267230 PMC1343481

[B28] Guzman J. N. Sanchez-Padilla J. Wokosin D. Kondapalli J. Ilijic E. Schumacker P. T.. (2010). Oxidant stress evoked by pacemaking in dopaminergic neurons is attenuated by DJ-1. Nature 468, 696–700. 10.1038/nature09536 21068725 PMC4465557

[B29] Heinsen H. Rüb U. Gangnus D. Jungkunz G. Bauer M. Ulmar G.. (1996). Nerve cell loss in the thalamic centromedian-parafascicular complex in patients with Huntington’s disease. Acta Neuropathol. 91, 161–168. 10.1007/s004010050408 8787149

[B30] Hernández-Flores T. Hernández-González O. Pérez-Ramírez M. B. Lara-González E. Arias-García M. A. Duhne M.. (2015). Modulation of direct pathway striatal projection neurons by muscarinic M4-type receptors. Neuropharmacology 89, 232–244. 10.1016/j.neuropharm.2014.09.028 25290553

[B31] Higley M. J. Soler-Llavina G. J. Sabatini B. L. (2009). Cholinergic modulation of multivesicular release regulates striatal synaptic potency and integration. Nat. Neurosci. 12, 1121–1128. 10.1038/nn.2368 19668198 PMC2733934

[B32] Holley S. M. Joshi P. R. Parievsky A. Galvan L. Chen J. Y. Fisher Y. E.. (2015). Enhanced GABAergic inputs contribute to functional alterations of cholinergic interneurons in the R6/2 mouse model of Huntington’s disease. eNeuro 2:e0008. 10.1523/ENEURO.0008-14.2015 26203463 PMC4507822

[B33] Kassubek J. Juengling F. D. Ecker D. Landwehrmeyer G. B. (2005). Thalamic atrophy in Huntington’s disease co-varies with cognitive performance: a morphometric MRI analysis. Cereb. Cortex 15, 846–853. 10.1093/cercor/bhh185 15459079

[B34] Kawaguchi Y. (1993). Physiological, morphological and histochemical characterization of three classes of interneurons in rat neostriatum. J. Neurosci. 13, 4908–4923. 7693897 10.1523/JNEUROSCI.13-11-04908.1993 PMC6576359

[B35] Kemp J. M. Powell T. P. (1971). The synaptic organization of the caudate nucleus. Philos. Trans. R. Soc. Lond. B Biol. Sci. 262, 403–412. 10.1098/rstb.1971.0103 4399121

[B36] Kosillo P. Zhang Y.-F. Threlfell S. Cragg S. J. (2016). Cortical control of striatal dopamine transmission via striatal cholinergic interneurons. Cereb. Cortex 26, 4160–4169. 10.1093/cercor/bhw252 27566978 PMC5066833

[B37] Lapper S. R. Bolam J. P. (1992). Input from the frontal cortex and the nucleus to cholinergic interneurons in the dorsal of the rat. Neuroscience 51, 533–545. 10.1016/0306-4522(92)90293-b 1488113

[B38] Lasser-Katz E. Simchovitz A. Chiu W.-H. Oertel W. H. Sharon R. Soreq H. (in press). Mutant α-synuclein overexpression induces stressless pacemaking in vagal motoneurons at risk in Parkinson’s disease. J. Neurosci. 1079–1116. 10.1523/JNEUROSCI.1079-16.2016 PMC6705667 28053029

[B39] Manza P. Hu S. Chao H. H. Zhang S. Leung H. C. Li C. S. R. (2016). A dual but asymmetric role of the dorsal anterior cingulate cortex in response inhibition and switching from a non-salient to salient action. Neuroimage 134, 466–474. 10.1016/j.neuroimage.2016.04.055 27126003 PMC4912860

[B40] Matsumoto N. Minamimoto T. Graybiel A. M. Kimura M. (2001). Neurons in the thalamic CM-Pf complex supply striatal neurons with information about behaviorally significant sensory events. J. Neurophysiol. 85, 960–976. 11160526 10.1152/jn.2001.85.2.960

[B41] Maurice N. Mercer J. Chan C. S. Hernandez-Lopez S. Held J. Tkatch T.. (2004). D2 dopamine receptor-mediated modulation of voltage-dependent Na^+^ channels reduces autonomous activity in striatal cholinergic interneurons. J. Neurosci. 24, 10289–10301. 10.1523/JNEUROSCI.2155-04.2004 15548642 PMC6730305

[B42] Menalled L. B. Sison J. D. Dragatsis I. Zeitlin S. Chesselet M. F. (2003). Time course of early motor and neuropathological anomalies in a knock-in mouse model of Huntington’s disease with 140 CAG repeats. J. Comp. Neurol. 465, 11–26. 10.1002/cne.10776 12926013

[B44] Minamimoto T. Hori Y. Kimura M. (2009). Roles of the thalamic CM-PF complex-Basal ganglia circuit in externally driven rebias of action. Brain Res. Bull. 78, 75–79. 10.1016/j.brainresbull.2008.08.013 18793702

[B43] Minamimoto T. Kimura M. (2002). Participation of the thalamic CM-Pf complex in attentional orienting. J. Neurophysiol. 87, 3090–3101. 10.1152/jn.00564.2001 12037210

[B45] Onali P. Olianas M. C. (2002). Muscarinic M4 receptor inhibition of dopamine D1-like receptor signalling in rat nucleus accumbens. Eur. J. Pharmacol. 448, 105–111. 10.1016/s0014-2999(02)01910-6 12144929

[B46] Parker P. R. L. Lalive A. L. Kreitzer A. C. (2016). Pathway-specific remodeling of thalamostriatal synapses in parkinsonian mice. Neuron 89, 734–740. 10.1016/j.neuron.2015.12.038 26833136 PMC4760843

[B47] Picconi B. Passino E. Sgobio C. Bonsi P. Barone I. Ghiglieri V.. (2006). Plastic and behavioral abnormalities in experimental Huntington’s disease: a crucial role for cholinergic interneurons. Neurobiol. Dis. 22, 143–152. 10.1016/j.nbd.2005.10.009 16326108

[B48] Plotkin J. L. Day M. Peterson J. D. Xie Z. Kress G. J. Rafalovich I.. (2014). Impaired TrkB receptor signaling underlies corticostriatal dysfunction in Huntington’s disease. Neuron 83, 178–188. 10.1016/j.neuron.2014.05.032 24991961 PMC4131293

[B49] Quik M. Zhang D. Perez X. A. Bordia T. (2014). Pharmacology and therapeutics role for the nicotinic cholinergic system in movement disorders; therapeutic implications. Pharmacol. Ther. 144, 50–59. 10.1016/j.pharmthera.2014.05.004 24836728 PMC4149916

[B50] Redman S. (1990). Quantal analysis of synaptic potentials in neurons of the central nervous system. Physiol. Rev. 70, 165–198. 2404288 10.1152/physrev.1990.70.1.165

[B51] Schwindt P. C. Crill W. E. (1995). Amplification of synaptic current by persistent sodium conductance in apical dendrite of neocortical neurons. J. Neurophysiol. 74, 2220–2224. 8592214 10.1152/jn.1995.74.5.2220

[B52] Shen W. Plotkin J. L. L. Francardo V. Ko W. K. D. K. D. Xie Z. Li Q.. (2015). M4 muscarinic receptor signaling ameliorates striatal plasticity deficits in models of L-DOPA-induced dyskinesia. Neuron 88, 762–773. 10.1016/j.neuron.2015.10.039 26590347 PMC4864040

[B53] Sidibe M. Smith Y. (1999). Thalamic inputs to striatal interneurons in monkeys: synaptic organization and co-localization of calcium binding proteins. Neuroscience 89, 1189–1208. 10.1016/s0306-4522(98)00367-4 10362307

[B54] Smith R. Chung H. Rundquist S. Maat-Schieman M. L. Colgan L. Englund E.. (2006). Cholinergic neuronal defect without cell loss in Huntington’s disease. Hum. Mol. Genet. 15, 3119–3131. 10.1093/hmg/ddl252 16987871

[B55] Song W. J. Tkatch T. Baranauskas G. Ichinohe N. Kitai S. T. Surmeier D. J. (1998). Somatodendritic depolarization-activated potassium currents in rat neostriatal cholinergic interneurons are predominantly of the A type and attributable to coexpression of Kv4.2 and Kv4.1 subunits. J. Neurosci. 18, 3124–3137. 9547221 10.1523/JNEUROSCI.18-09-03124.1998 PMC6792663

[B56] Strichartz G. R. (1973). The inhibition of sodium currents in myelinated nerve by quaternary derivatives of lidocaine. J. Gen. Physiol. 62, 37–57. 10.1085/jgp.62.1.37 4541340 PMC2226105

[B57] Tepper J. M. Bolam J. P. (2004). Functional diversity and specificity of neostriatal interneurons. Curr. Opin. Neurobiol. 14, 685–692. 10.1016/j.conb.2004.10.003 15582369

[B58] Thomas T. M. Smith Y. Levey A. I. Hersch S. M. (2000). Cortical inputs to m2-immunoreactive striatal interneurons in rat and monkey. Synapse 37, 252–261. 10.1002/1098-2396(20000915)37:4<252::AID-SYN2>3.0.CO;2-A 10891862

[B59] Threlfell S. Lalic T. Platt N. J. Jennings K. A. Deisseroth K. Cragg S. J. (2012). Striatal dopamine release is triggered by synchronized activity in cholinergic interneurons. Neuron 75, 58–64. 10.1016/j.neuron.2012.04.038 22794260

[B60] Wang L. Zhang X. Xu H. Zhou L. Jiao R. Liu W.. (2014). Temporal components of cholinergic terminal to dopaminergic terminal transmission in dorsal striatum slices of mice. J. Physiol. 592, 3559–3576. 10.1113/jphysiol.2014.271825 24973407 PMC4229348

[B61] Wastek G. J. Yamamura H. I. (1978). Biochemical characterization of the muscarinic cholinergic receptor in human brain: alterations in Huntington’s disease. Mol. Pharmacol. 14, 768–780. 152407

[B62] Wilson C. J. Chang H. T. Kitai S. T. (1990). Firing patterns and synaptic potentials of identified giant aspiny interneurons in the rat neostriatum. J. Neurosci. 10, 508–519. 2303856 10.1523/JNEUROSCI.10-02-00508.1990 PMC6570144

[B63] Yan Z. Surmeier D. J. (1996). Muscarinic (m2/m4) receptors reduce N^-^ and P-type Ca^2+^ currents in rat neostriatal cholinergic interneurons through a fast, membrane-delimited, G-protein pathway. J. Neurosci. 16, 2592–2604. 8786435 10.1523/JNEUROSCI.16-08-02592.1996 PMC6578763

[B64] Zuccato C. Valenza M. Cattaneo E. (2010). Molecular mechanisms and potential therapeutical targets in Huntington’s disease. Physiol. Rev. 90, 905–981. 10.1152/physrev.00041.2009 20664076

